# Minimum-noise production of translation factor eIF4G maps to a mechanistically determined optimal rate control window for protein synthesis

**DOI:** 10.1093/nar/gkw1194

**Published:** 2016-12-07

**Authors:** Xiang Meng, Helena Firczuk, Paola Pietroni, Richard Westbrook, Estelle Dacheux, Pedro Mendes, John E.G. McCarthy

**Affiliations:** 1 Warwick Integrative Synthetic Biology Centre (WISB) and School of Life Sciences, University of Warwick, Gibbet Hill, Coventry CV4 7AL, UK; 2 Center for Quantitative Medicine, UConn Health, 263 Farmington Avenue, CT 06030-6033, USA

## Abstract

Gene expression noise influences organism evolution and fitness. The mechanisms determining the relationship between stochasticity and the functional role of translation machinery components are critical to viability. eIF4G is an essential translation factor that exerts strong control over protein synthesis. We observe an asymmetric, approximately bell-shaped, relationship between the average intracellular abundance of eIF4G and rates of cell population growth and global mRNA translation, with peak rates occurring at normal physiological abundance. This relationship fits a computational model in which eIF4G is at the core of a multi-component–complex assembly pathway. This model also correctly predicts a plateau-like response of translation to super-physiological increases in abundance of the other cap-complex factors, eIF4E and eIF4A. Engineered changes in eIF4G abundance amplify noise, demonstrating that minimum stochasticity coincides with physiological abundance of this factor. Noise is not increased when eIF4E is overproduced. Plasmid-mediated synthesis of eIF4G imposes increased global gene expression stochasticity and reduced viability because the intrinsic noise for this factor influences total cellular gene noise. The naturally evolved eIF4G gene expression noise minimum maps within the optimal activity zone dictated by eIF4G's mechanistic role. Rate control and noise are therefore interdependent and have co-evolved to share an optimal physiological abundance point.

## INTRODUCTION

It is estimated that more than 76% of yeast's total cellular energy budget is committed to protein synthesis (1). Moreover, the control of protein synthesis is closely linked to growth capacity (and thus competitiveness) and has accordingly been honed by selective pressures over hundreds of millions of years. Studies in microorganisms have suggested that, despite the complexity of genetic and metabolic control networks, the relationship between growth and the biosynthetic capacity of the translation machinery seems to follow relatively simple principles (2). Imprecision in the control of protein synthesis is a potential threat to organism survival; equally, gene expression noise generated via the translation machinery can be expected to influence the viability of individual cells. To place this into context, recent modeling work has estimated the effective cost of noise as equivalent to up to 25% of overall yeast fitness (3).

**Figure 1.**
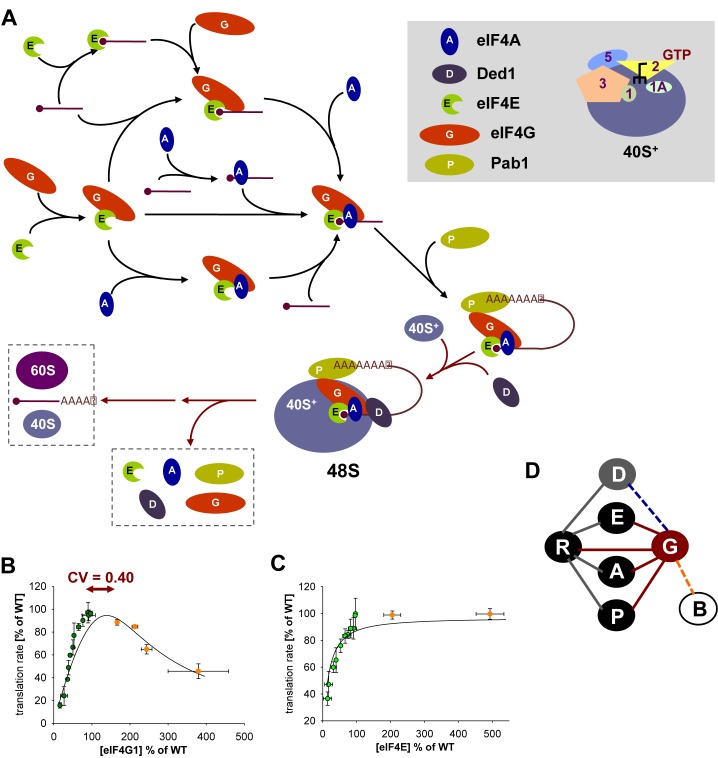
The role of eIF4G in the translation initiation pathway. There are potentially multiple routes to formation of the 48S pre-initiation complex. Here we illustrate alternative paths to formation of the cap-binding complex on capped mRNA, followed by further steps *en route* to 40S subunit recruitment (**A**). In the interest of clarity, we represent the 40S ribosomal subunit plus eIF1, eIF1A, Met-tRNA_i_-eIF2-GTP, eIF3 and eIF5 as 40S^+^ (see key in the grey panel). Further reaction steps shown here represent the disassembly of the cap-binding complex and the combination of scanning to the AUG start codon and joining of the 60S ribosomal subunit. (**B)** Experimental data from previous work that used a chromosomal *tet07* construct to delineate the relationship between eIF4G abundance (below the wild-type level; green data points) and global translation rate combined with new data exploring this relationship in the region >100% eIF4G abundance (orange data points). The fit (black line) has been generated by allowing the pathway model (Supplementary Figure S2) to generate a fit to the experimental data. Overexpression was achieved by replacing the natural promoter P*_TIF4631_* with P*_TDH3_*, P*_TEF1_*, P*_PAB1_* or P*_TRP1_* upstream of *TIF4631*. The x-axis shows the abundance of eIF4G as a percentage of the average wild-type abundance of this factor. Values on the y-axis are shown as percentage of the average wild-type global protein synthesis rate. For comparison, the magnitude of the coefficient of variance for eIF4G::GFP (total noise, as determined by flow cytometry) is also represented on the plot. (**C)** Equivalent plot and computational model fit for eIF4E. (**D)** An interaction map with nodes representing the translation factors eIF4A, eIF4B, eIF4E, eIF4G, Ded1 and Pab1 (see key in grey panel), and edges representing the known possible interactions. mRNA is represented by R. The multiple interactions of eIF4G (with R, A, E and P) that are on the main assembly pathway are highlighted in red. Dashed lines indicate (transient) interactions with eIF4B and Ded1.

A critical step in cap-dependent translation initiation in eukaryotes involves association of the eukaryotic translation initiation factor eIF4G with the cap-binding protein eIF4E (encoded by *CDC33*; Figure 1A). eIF4G helps recruit the ribosomal 43S complex (comprising the 40S subunit plus the Multi-Factor-Complex (MFC) factors eIF1, Met-tRNA^Met^.eIF2.GTP, eIF3 and eIF5, together with eIF1A) to the 5′end of mRNA (4). eIF4E and eIF4G, together with the DEAD-box helicase eIF4A (encoded by *TIF1* and *TIF2*), also form the cap-binding complex eIF4F (Figure 1A), and eIF4G-Pab1 interactions are capable of mediating interactions between the 5′ and 3′ ends of mRNA (5). The high affinities between eIF4E, eIF4G and mRNA suggest that these three components readily form a core complex *in vivo* (6–8), whereby eIF4G is thought to act as a molecular scaffold upon which this, and even higher order complexes, are built. eIF4G exists in two isoforms in *S. cerevisiae* (eIF4G1/eIF4G2, encoded by *TIF4631* and *TIF4632*, respectively), in mammalian cells (eIF4GI/eIF4GII), and in plants (4). There is a further essential DEAD-box helicase, called Ded1, that can associate with the cytoplasmic (and nuclear) cap-binding complex. This protein promotes translation initiation but the mechanism of its action is unclear (9,10).

Translation initiation involves cycles of progressive stoichiometric assembly (and disassembly) of intermediate complexes (Figure 1A). A recent comprehensive rate control study (11) revealed that control of the rate of protein synthesis, at least under exponential growth conditions, is distributed primarily over a subset of components of the translation machinery. eIF4G exerts stronger rate control over yeast protein synthesis *in vivo* than the cap-binding protein eIF4E, a factor that has long been regarded as a key controlling factor in eukaryotic protein synthesis. This high flux-control value is consistent with the critical molecular ‘scaffolding’ function of eIF4G, a factor that interacts with multiple partners. eIF4G is one of the least abundant translation factors in yeast, with an average intracellular abundance approximately forty times lower than that of the elongation factor eEF1A, and ten-fold lower than that of eIF4A (the most abundant initiation factor), yet both eIF4G and eIF4A exert comparatively strong rate control over protein synthesis (11). There is evidence that the intracellular control of eIF4G activity has wider significance. For example, reduced abundance can extend the lifespan of yeast and nematodes, and overabundance is correlated with certain cancers in humans (12).

Previous genetic and biochemical studies of translational control have generally reported mechanistic and control data averaged over many millions of cells. Our focus in this study is the role of eIF4G at the single cell level. In a wider context, there are many intracellular machineries that feature key (scaffolding) proteins which bind multiple other components (examples include the pheromone signaling pathway (13), ribosome biogenesis (14), and proteasome assembly (15)). Therefore studying molecular noise and single-cell functionality in relation to eIF4G is of broader relevance.

Here we observe that, while the average abundance of eIF4G across a population of cells is quantitatively aligned with the mechanistic requirements of the protein synthesis pathway, individual cells manifest significant variation (noise) in the numbers of molecules of eIF4G mRNA and of eIF4G protein. Given the powerful role of eIF4G as a rate-controlling factor and the mechanistic features constraining its optimum abundance range, this heterogeneity has the potential to act as a driver of variation in protein synthesis capacity between individual cells, thus generating extrinsic noise that impacts on the entire cellular system. We explore the relationship that has evolved in yeast between optimizing average population-level protein synthesis efficiency and the observed level of cell-to-cell heterogeneity for expression of the eIF4G gene. Overall, this provides insight into the relationship between mechanistic features of the initiation phase of protein synthesis and the noise parameters for the translation machinery in individual cells.

## MATERIALS AND METHODS

### Strain construction and analysis

The *Saccharomyces cerevisiae* strains used in this paper are summarized in a table in the Supplementary Data section. Unless otherwise stated, standard methods were used to construct the yeast strains, all of which are based on PTC41. For the construction of genomic GFP gene fusions, a cassette comprising the yeast enhanced GFP (yeGFP) gene and a selective marker was chromosomally integrated and fused to the C termini of the respective target genes. For the construction of cassettes combining the P*_TEF1_* promoter with a range of stem-loop structures, the P*_TEF1_* promoter plus a selective marker were amplified using specific primers containing the stem-loop sequences in the flanking region. Each of the resulting cassettes was chromosomally integrated upstream of the coding region of the target gene. The *tetO7* strains created for characterization of the eIF4G abundance versus protein synthesis relationship were constructed and analysed as described previously (11). A range of promoters were also used to boost the rate of synthesis of eIF4G1 to yield abundance levels above the physiological norm (Figure 1B; Supplementary Data). Mass spectrometric analysis of translation factor concentrations was performed (in triplicate) on a Thermo Scientific™ TSQ Quantiva™ Triple Quadrupole Mass Spectrometer via the selected reaction monitoring (SMP) approach using Skyline software (16). Signal strengths were compared to those obtained in earlier, internally calibrated, estimations of protein abundance (11). Details of the mass spectrometry methods and data are provided in the Supplementary Data section.

### Cell-to-cell heterogeneity measurements

One day prior to a batch of experiments, single colonies from each of the strains were picked and grown overnight in YNB (plus amino acids) medium with shaking at 30°C. The following morning, cells were diluted to give an optical density at 600 nm (OD_600_) of ∼0.2. Diluted cultures were then grown to mid-exponential phase (OD_600_ ∼ 0.6) with continued shaking at 30°C. smFISH (17) was performed on fixed, mid-exponential-phase, yeast cells using (Quasar 570- and Quasar 670-tagged) probes (listed in the Supplementary Data section) targeted to multiple regions of the respective gene sequences. For flow cytometry, a mid-log phase culture from each strain was subjected to sonication at 50–60 Hz for ∼15 s in order to separate any cell aggregates. After sonication, sterilized PBS was added to each tube for flow cytometry measurements. To minimize variation due to delays between sample delivery and measurements, different strains were prepared for flow cytometry at intervals of 15 min. Yeast cells synthesizing GFP fusion proteins were illuminated using a 488 nm laser, and fluorescence was collected through 505 nm long-pass and 530/30 nm band-pass filters on a BD Fortessa X20 flow cytometer. The data were recorded using the ‘Area’ option. Flow cytometry data were exported from the acquisition program (FACSDiva) in the FCS3.0 format with a data resolution of 2^18^. A custom R programme was written (using flowCore, flowViz and flowDensity Bioconductor packages) to calculate statistics for each file. For calculating the coefficients of variation (CV values), cytometry files were processed as follows:

1. The first second, and last 0.2 s, of data were removed to minimize errors due to unstable sample flow through the cytometer.
2. Thresholds of 40 000–100 000 and 10 000–90 000 for the FSC and SSC gates, respectively, were typically used to limit the influence of cellular debris and aggregated cells.
3. For the remaining data, the FSC and SSC values of the highest density centre of the FSC–SSC scatterplot were calculated, and the distance of the *i*th sample to the centre was determined:
  \begin{equation*}{\rm{Distance}}\,{\rm{i}} = \surd \left( {{{\left( {{\rm{FSC}}\,{\rm{i}} - {\rm{FSC}}\,{\rm{centre}}} \right)}^2}{\rm{ + }}{{\left( {{\rm{SSC}}\,{\rm{i}} - {\rm{SSC}}\,{\rm{centre}}} \right)}^2}} \right)\end{equation*}
4. The GFP data within the radius were used to calculate the coefficient of variation: CV = σ / μ

The CV values for ratios between GFP-tagged proteins and mCherry-tagged eIF4A2 (Figure 3) were calculated according to the formula:

\begin{equation*} Nois{e^2} = \frac{{\left\langle {{{(GFP - mCherry)}^2}} \right\rangle }}{{2\langle GFP\rangle \langle mCherry\rangle }} \end{equation*}

as described previously (18).

### Growth competition method

Growth competition experiments were conducted to detect small differences in growth rate between distinct strains: WT, P*_TIF4631_TIF4631::GFP* and P*_TEF1_*(M2)*TIF4631::GFP*. Single colonies from each of the strains were picked and grown overnight with shaking at 30°C. The following morning, cells were diluted to give an optical density at 600 nm (OD_600_) of ∼0.1. Diluted cultures were then mixed individually in a 1:1 ratio with an isogenic competitor strain (*lys2::kanMX*, in which the *LYS2* gene in the WT strain PTC41 was disrupted by the *kanMX* gene). The accuracy of strain mixing was controlled via OD measurements. Then each mixed culture was grown consistently at near mid-log phase (OD_600_ ∼0.6) by repeated dilutions for approximately 98 hours with shaking at 30°C. The proportion of each strain in a mixed population was assessed by plating on (selective) growth media: YPD, YPD + G418 (selection against tested strains), and YNB-Lys (selection against the control *lys2::kanMX* strain) prior to (*t*_0_) and after (*t*_98_) for the mid-log phase culture. Colony numbers (No.) were counted for each of the plates in order to determine the percentage of each test strain remaining in the total population of cells. The percentage of each tested strain in a population was calculated using the following formula:

\begin{eqnarray*} \begin{array}{*{20}{l}} {\% \ \,{\rm{of}}\,{\rm{population}}}\\ { = \ \frac{{{\rm{No}}.\,{\rm{tested}}\,({{\rm{t}}_{98}})/{\rm{No}}.\,{\rm{tested}}\,({{\rm{t}}_0})}}{{\ {\rm{No}}.\,{\rm{tested}}\,({{\rm{t}}_{98}})/{\rm{No}}.\,{\rm{tested}}\,({{\rm{t}}_0}) + {\rm{No}}.\,{\rm{competitor}}\,({{\rm{t}}_{98}})/{\rm{No}}.\,{\rm{competitor}}\,({{\rm{t}}_0})}}} \end{array} \end{eqnarray*}

### Computational rate control model of cap complex assembly

The computational model comprises a random-order binding scheme where eIF4G and mRNA act as scaffolds, and where eIF4A, eIF4E, Ded1 and Pab1 can bind both scaffolds. This reaction scheme was created as a set of rules with the software BioNetGen (19) which generates an SBML model with the entire reaction network consisting of 1744 reactions and 211 chemical species (note that these reactions are irreversible and correspond to 872 reversible binding reactions). This model of cap-binding complex formation was then imported to the software COPASI (20) and complemented with two extra reactions to mimic the rest of translation: one reaction where the complex eIF4A:eIF4E:eIF4G:Ded1:Pab1:mRNA binds the 43S complex, creating an elongating ribosome and free forms of eIF4A, eIF4E, eIF4G, Ded1 and Pab1; the second reaction where the elongating ribosome dissociates to a free 40S ribosomal subunit, free peptide and free mRNA. The role of these two steps is to allow the system to reach a steady state where mRNA is continuously being incorporated into ribosomal complexes and peptides are being produced, to match the experimental scenario where translation is operating in a quasi-steady state. The final model thus has 1746 reactions and 215 species and is supplied in a supplementary file containing the initial BioNetGen rules, and the final model in SBML and COPASI formats. The final model was simulated, and its parameters estimated from the experimental data, using the software COPASI. This represents the model as a set of 213 simultaneous ordinary differential equations, but which only contains 30 parameters given that no binding cooperativity was considered (28 parameters for the random-order cap-binding complex and 2 for the two reactions representing the remaining steps of translation). Parameter estimation was carried out in a sequence of 10 steps, alternating the Hooke-Jeeves algorithm (21) with the Particle Swarm algorithm (22). A more detailed description of the model and how it was applied is given in the Supplementary Data section.

## RESULTS

### Optimal global translation rate within a narrow eIF4G activity range

Previous work demonstrated that global protein synthesis in exponentially growing cultures of yeast cells is highly sensitive to reductions in the intracellular concentration of eIF4G (11). In this earlier study, eIF4G1 activity was modulated incrementally (via ‘titration’ with doxycycline) using a chromosomal *tet07* regulatory construct in a strain deleted for eIF4G2 (*tif4632Δ*). The rate control coefficient (R^J^_1_; change of the rate of global protein synthesis as a function of the change in intracellular abundance of the specific translation factor) was taken from the slope of the plot in the near-physiological range (80–100% of the wild-type average global protein synthesis rate). Moreover, all three cap-complex proteins (eIF4A, eIF4E and eIF4G) were found to manifest strong R^J^_1_ values, thus emphasising the key role of the cap-binding complex in the translation pathway.

To clarify the contribution of the second eIF4G isomer, we performed the equivalent rate control study for eIF4G2 in a strain lacking eIF4G1 (*tif4631Δ*). It has been shown previously that eIF4G2 is capable of compensating for the absence of eIF4G1, provided that *TIF4632* is expressed to a sufficiently high level (23). Here, we found that eIF4G2 has a very similar R^J^_1_ value to that of eIF4G1 (Supplementary Figure S1). In other words, the two isomers are equivalent in terms of their respective abilities to exert rate control during exponential growth in yeast, a result that informs the modeling analysis performed in this study.

Variations in gene expression steps can lead to the intracellular abundance of a protein in any given cell being either below, or in excess of, the average abundance measured by standard biochemical methods for cell populations. To help inform our understanding of the single cell biology of eIF4G-mediated translational control, we integrated a range of alternative promoters upstream of *TIF4631* (Figure 1B) in order to explore the relationship between average eIF4G abundance and global protein synthesis and growth in the region above wild-type abundance. Combining the resulting new overexpression data with earlier results obtained for the region below wild-type abundance of eIF4G (11), we observe that the relationship of the global mRNA translation rate to the average intracellular abundance of eIF4G describes an asymmetric bell-shaped curve centered around the physiological level (determined previously to be ∼22 000 molecules per cell of the two eIF4G isomers combined). In contrast, overproduction of eIF4E (24; Figure 1C) or of eIF4A has comparatively little effect on translation or growth in yeast.

In order to obtain insight into why protein synthesis is inhibited strongly both by an excess, as well as by an insufficiency, of intracellular eIF4G, we established a computational model of the assembly of the cap-binding complex on capped mRNA (Materials and Methods section; Supplementary Figure S2) based on the interaction relationships summarized in Figure 1. The model represents the assembly of the cap-binding complex proteins eIF4A, eIF4E and eIF4G together with the 5′end of the mRNA (some of the possible reaction steps are illustrated in Figure 1A). It also includes Ded1, a further essential DEAD-box helicase that is required for translation initiation (9). Strong overexpression of Ded1 drives formation of a translationally repressed Ded1–eIF4F–mRNA complex that accumulates in stress granules (25). Since the repressed complex is not thought to form in wild-type cells under conditions of exponential growth on glucose, it does not feature in our model. Moreover, yeast has a non-essential eIF4B factor that is thought to promote binding between eIF4A and eIF4G by somehow stabilizing a specific conformation of the eIF4G HEAT domain (26). However, this protein has no effect on the binding between recombinant eIF4A and eIF4G *in vitro* (26), and it seems unable to form a heterotrimeric complex together with these two factors. eIF4B binding is likely to be transient and it has an *in vivo* rate control coefficient in the near-physiological abundance range that is far lower than those of the other eIF4F complex factors (11). For the sake of simplicity, we have therefore not included eIF4B in the model at this stage (Figure 1D and Supplementary Figure S2).

A random assembly model (Figure 1A; Supplementary Figure S2), which is consistent with previous biochemical, biophysical and genetic investigations (see, e.g. (8, 27–29)), predicts that the required binding of eIF4G to multiple other factors to build the cap-binding complex constrains the optimal functional concentration range of this factor, thus providing a good fit to the experimentally observed data (Figure 1B). The observed asymmetric bell-shaped profile is reminiscent of the so-called ‘prozone’ effect reported in early work on the formation of the three-dimensional lattice of interactions between antigen and antibody (30,31). Equally, the same model correctly predicts no comparable inhibitory behavior arising from excess levels of eIF4E (Figure 1C).

### Cell-to-cell heterogeneity in mRNA abundance

**Figure 2.**
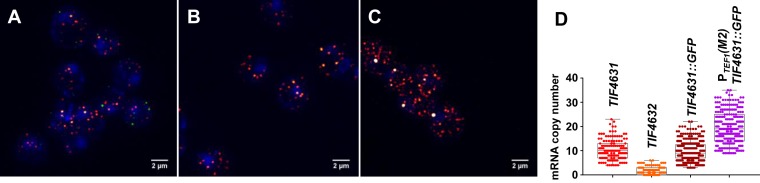
Cell-to-cell heterogeneity of eIF4G mRNA. smFISH experiments for simultaneous labelling of non-fused *TIF4631* (red) and *TIF4632* (green) mRNAs (**A**), P*_TIF4631_TIF4631::GFP* mRNA (**B**), and P*_TEF1_*(M2)*TIF4631::GFP*-encoded mRNA (**C**). Distributions of mRNA counts per cell across cell populations (**D**) reveal CV values for the mRNA species (see main text and Supplementary Figure S3). All cells were also treated with the nuclear stain DAPI.

We next determined whether eIF4G gene expression manifests marked stochasticity, since cell viability can be expected to be sensitive to both upward and downward variations in the abundance of this protein. Moreover, fluctuations in gene expression generally tend to be more significant at lower intracellular transcript levels (32). We started our characterization of the heterogeneity in the synthesis of this factor using smFISH (single molecule Fluorescence *In Situ* Hybridization). eIF4G mRNA copy number was determined using fluorescently tagged mRNA-specific probes targeted to *TIF4631* and *TIF4632* mRNAs, respectively (Figure 2A and D). Our results indicate that the average intracellular eIF4G mRNA abundance values are 11 ± 4 (*TIF4631*; total population CV = 0.36) and 2 ± 2 (*TIF4632*; total population CV = 1.00), respectively, whereby the copy numbers for the respective isomer mRNA species in each cell are at best weakly correlated (Supplementary Figure S3). The *CDC33* and *TIF1/TIF2* mRNA counts per cell [14±5 (CV = 0.36) and 57±10 (CV = 0.18), respectively; Supplementary Figure S3] reflect the large difference in intracellular concentrations of eIF4E and eIF4A (11), and we note that, overall, the mRNA CV values follow the order: *TIF4632* (eIF4G2) > *TIF4631* (eIF4G1) ≥ *CDC33* (eIF4E) > *TIF1*/*TIF2* (eIF4A). Collectively, the mRNA abundance values exceed those estimated previously using high-density oligonucleotide arrays (33,34), a finding that is relevant to our analysis of gene expression noise. This apparent discrepancy may be attributable to an underestimation of the total number of all mRNA species in yeast, to which many hybridization-based abundance estimates have been normalized (34).

### Cell-to-cell variation in cap-complex protein abundance

Previous studies have generated conflicting conclusions regarding the relationship between mRNA copy number and variation in the single-cell abundance of an encoded protein (35,36). Given that the degree of cell-to-cell eIF4G heterogeneity can be expected to impact on the protein synthesis capacity of individual cells, we characterized the noise for the two isomers at the protein level. We constructed *GFP* gene chromosomal fusions of *TIF4631* and *TIF4632* in which the full reading frames (and the functionalities) of the respective genes were maintained. This allowed us to study the distribution of the encoded eIF4G::GFP fusion proteins across populations of exponentially growing yeast cultures using flow cytometry. In separate experiments, we found that the *TIF4631::GFP* and *TIF4632::GFP* mRNAs manifest very similar intracellular content distributions to those of the unfused mRNAs [e.g. *TIF4631::GFP* (10±4; Figure 2B) vs *TIF4631* (11 ± 4; Figure 2A)].

**Figure 3.**
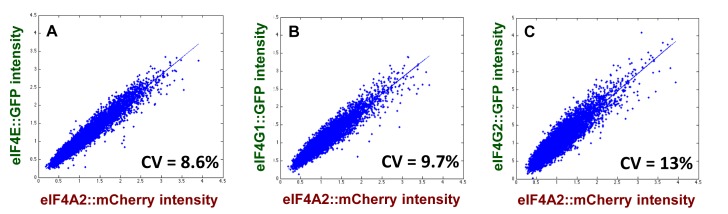
Cell-to-cell heterogeneity in the ratios between cap-complex translation factors. Flow cytometry data were collected for GFP fusions with eIF4E (**A**), eIF4G1 (**B**) and eIF4G2 (**C**) using, in each strain, a fusion between mCherry and eIF4A2 as a reference. The plots reflect the cell-to-cell variation in the ratio between eIF4A2 and the other three factors. The CV values were calculated according to a formula given in the Materials and Methods section.

Overall gene expression noise is generally regarded as comprising components that are intrinsic (specific to a given gene in a single cell) and extrinsic (global differences between cells in terms of the gene expression environment). Intrinsic and extrinsic noise sources can in principle be distinguished by comparing two (physiologically non-essential and non-perturbing) reporter genes that are identically regulated (37). Taking into account the sensitivity of cell growth to translation factor abundance, we have adopted a modified version of this strategy in which we determine the expression ratio between two distinct translation factor genes that have been tagged with different fluorescent reporters. We determined the intracellular ratio between the intensity of eIF4A2::mCherry and each of the GFP fusions with the other cap complex factors (Figure 3A–C). We used this ‘reference construct’ because we have shown previously that yeast exponential growth is relatively insensitive to expression changes if these affect only one of either of the two eIF4A alleles (*TIF1* and *TIF2*; 11). The (uncorrelated) cell-to-cell variation in the ratios between eIF4G, eIF4E and eIF4A followed the trend eIF4G2 > eIF4G1 > eIF4E.

**Figure 4.**
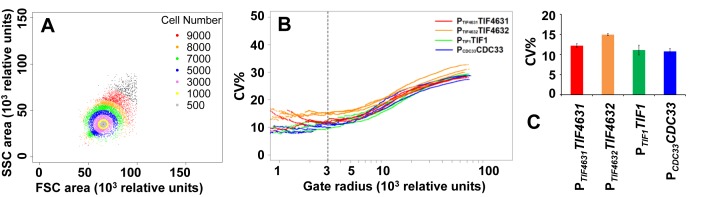
Estimation of the intrinsic noise for cap-complex translation factors. An SSC versus FSC plot (**A**) illustrates the procedure used to identify subsets of cells that are increasingly homogeneous in terms of factors that contribute to extrinsic noise. A plot of CV versus gate radius (**B**) reveals the approximation to a minimum CV at lower gate radii for repeated experiments (here we show three for each, colour-coded) with genomically integrated fusions of *GFP* with *TIF4631, TIF4632, TIF1* and *CDC33*. The average noise values (coefficients of variance) and corresponding standard deviations for these four types of genomic construct are represented in a bar graph (**C**).

Much of the extrinsic component of gene expression noise is related to heterogeneity in size, shape and cell-cycle stage in a cell population (see, e.g. (38,39)). The use of flow cytometry gating based on forward (FSC) and side (SSC) scatter parameters to limit the influence of such heterogeneity on the measured variation in protein abundance offers an alternative approach to minimising the contribution of extrinsic noise in the estimated CV value (40). We have applied gating analysis to the data obtained with the translation factor GFP fusions in an otherwise wild-type strain background (Figure 4A and B). Consistent with the gene expression ratio measurements (Figure 3), these experiments indicate a noise trend of eIF4G2 > eIF4G1 > eIF4E ∼ eIF4A. Comparison of the abundance ratio values (Figure 3) with the values derived from the gating method (Figure 4) for eIF4G2 (13% versus 15.7%), eIF4G1 (9.7% vs 11.6%) and eIF4E (8.6% versus 10.9%) indicates that both methods provide a good indication of the intrinsic noise for the respective translation factors. Indeed, the percentage change in CV value derived from the two types of method (taking eIF4G2 as the reference, normalized to 1.0) are strikingly close: the abundance ratio method yields values of 1.0 (eIF4G2), 0.75 (eIF4G1) and 0.66 (eIF4E), while the gating method yields values of 1.0 (eIF4G2), 0.73 (eIF4G1) and 0.69 (eIF4E). The cell-to-cell variance for eIF4G2 alone is greater than for eIF4G1 but, taking into account the isomers’ respective abundance levels, the observed total noise for both factors together (Table 1) suggests that the individual noise contributions combine additively.

**Table 1.** Summary of genomic GFP fusion expression data

Construct/genotype	Total		Gated	
	GFP mean	CV%	CV%	GFP mean
**P*_TIF4631_TIF4631::GFP***	1250 ± 63.0	30.7 ± 0.8	11.6 ± 1.2	869 ± 57.9
**P*_TIF4632_TIF4632::GFP***	464 ± 22.5	33.7 ± 1.0	15.7 ± 0.9	324 ± 25.8
**P*_TIF4632_TIF4632::GFP* (*TIF4631Δ*)**	495 ± 5.5	35.6 ± 1.2	18.6 ± 1.4	358 ± 10.3
**P*_TIF4631_TIF4631::GFP* (*TIF4632Δ*)**	1170 ± 29.1	31.7 ± 0.6	11.8 ± 0.7	823 ± 82.9
**P*_TIF4631_TIF4631::GFP^PEST^***	969 ± 21.5	37.3 ± 0.9	16.0 ± 2.2	645 ± 18.6
**P*_TIF4631_TIF4631::GFP* / P*_TIF4632_TIF4632::GFP***	1570 ± 43.7	33.0 ± 0.4	12.7 ± 0.7	1140 ± 22.0
**P*_TEF1_*(M2)*TIF4631::GFP***	1310 ± 54.0	32.8 ± 1.1	11.6 ± 0.6	872 ± 42.6
**P*_TEF1_*(M2b)*TIF4631::GFP***	1075 ± 33.7	33.2 ± 0.4	12.1 ± 0.8	727 ± 27.8
**P*_TEF1_*(M3)*TIF4631::GFP***	583 ± 5.5	39.9 ± 0.1	19.1 ± 1.5	369 ± 3.6
**P*_TEF1_*(4)*TIF4631::GFP***	2600 ± 121	41.0 ± 0.7	13.7 ± 0.4	1450 ± 135
**P*_TIF1_TIF1::GFP***	3014 ± 112	32.5 ± 1.1	11.1 ± 1.3	2070 ± 270
**P*_CDC33_CDC33::GFP***	2960 ± 60.1	30.8 ± 0.6	10.9 ± 0.9	1981 ± 118
**P*_TEF1_CDC33::GFP***	15400 ± 898	32.2 ± 1.1	12.1 ± 0.8	10500 ± 698
**P*_TEF1_TIF4631* / P*_TIF4632_TIF4632::GFP***	540 ± 1.5	33.6 ± 0.6	16.9 ± 0.3	368 ± 11.6
P_TIF4631_TIF4631::GFP* [pP*_TRP1_TIF4631]*	1160 ± 56.6	37.1 ± 0.3	13.8 ± 0.1	777 ± 78.2
P_TIF4631_TIF4631::GFP* [pP*_TRP1_TIF4632]*	1110 ± 45.8	41.9 ± 1.7	14.4 ± 0.4	703 ± 30.8

The names of the constructs listed here indicate the identity of the promoter and of the gene in each case. All constructs are genomic integrations unless

preceded by *p* (to indicate plasmid) within brackets. Measures used to reduce *TIF4631* expression include fusion with a destabilizing PEST sequence, insertion of inhibitory stem-loops (M2, M2b, M3; Supplementary Figure S5) into the 5′UTR, and shortening of the 5′UTR to 4 nucleotides (construct(4)). Genomic *TIF4631* was deleted in the strains carrying plasmids (marked with an asterisk).

### Intrinsic vs extrinsic gene expression noise in the translation machinery

An important aspect of stochasticity associated with translation machinery components is the potential for noise generated during their intracellular synthesis to contribute to the global noise level in the cell. We set out to obtain a more complete understanding of this potential effect via experiments in which we examined the (indirect) effects of modulation of the expression of one translation factor gene on the expression noise of another. First of all, we found that the noise value for a genomically encoded eIF4G2::GFP fusion is increased in a *TIF4631Δ* strain (Table 1); here, the reduction in combined eIF4G activity attributable to the loss of eIF4G1 resulted in enhanced noise. In a complementary experiment, we observed that genomic overproduction of a non-tagged eIF4G1 (overexpression of *TIF4631* from the strong P*_TEF1_* promoter) resulted in increased noise in the expression of genomic *TIF4632::GFP* (Table 1).

In a second type of experiment, we characterized the impact of the inherently more variable rate of plasmid-directed synthesis of a translation factor on the expression noise of a genomic GFP fusion. We used stable low-copy centromeric plasmids for these experiments. Transformation of a *TIF1Δ* strain with a plasmid carrying the natural *TIF1* promoter plus *TIF1* reading frame created a strain in which genomically encoded eIF4A::GFP synthesis (*TIF4632::GFP*) manifested higher stochasticity (Supplementary Figure S4). Another manifestation of this type of effect is evident in the increased expression noise of genomically integrated *TIF4631::GFP* in a *TIF4631Δ* strain transformed with a plasmid carrying P*_TRP1_TIF4631* or P*_TRP1_TIF4632* (Table 1). As with the *TIF1* plasmid experiment described above, this shows that the greater variability in copy number of the plasmid-borne translation factor gene influences the cell-to-cell heterogeneity of the genomic GFP fusion indirectly by increasing the total stochasticity of the translation machinery.

### The relationship between stochasticity, rate control and fitness

**Figure 5.**
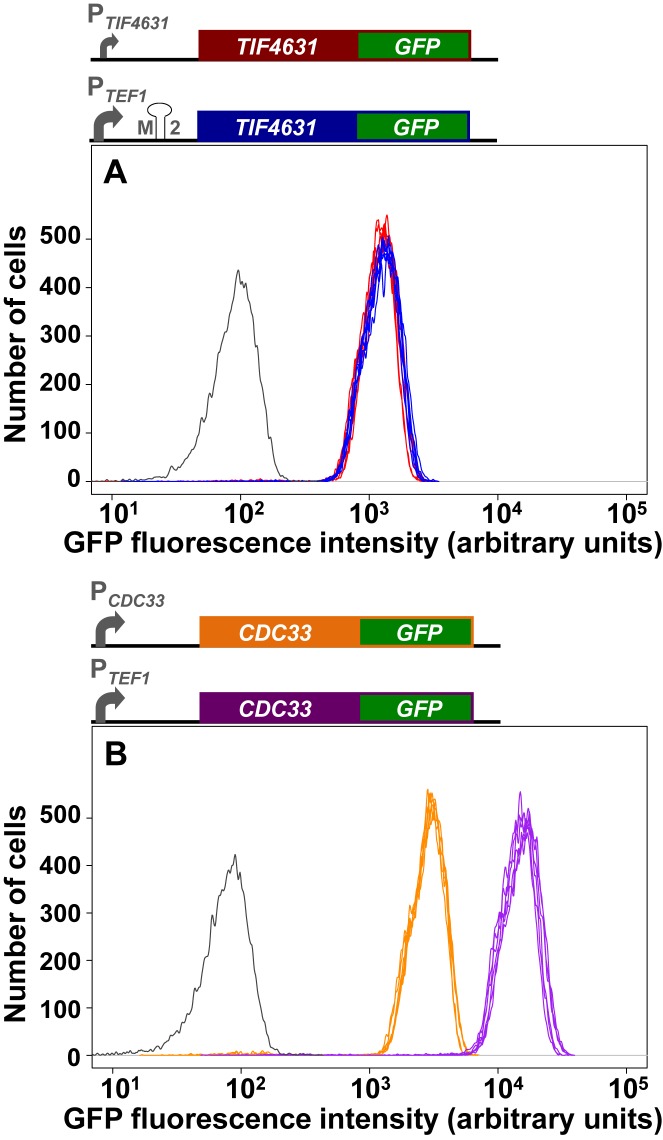
Fluorescence profiles for GFP fusions generated using alternative promoters. Comparison of flow-cytometry data for expression of *TIF4631::GFP* using the natural promoter P*_TIF4631_*combined with the natural *TIF4631* 5′UTR and the much stronger P*_TEF1_* promoter combined with a 5′UTR containing the M2 stem-loop (**A**). (**B**) Flow cytometry data for genomically integrated *CDC33::GFP* expressed either from the natural promoter P*_CDC33_* or from P*_TEF1_*, both constructs using the natural *CDC33* 5′UTR. For clarity, we show only the outlines (colour-coded) of flow cytometry distributions for six repeats of each experiment.

Control over the expression of a gene that plays a critical role in global gene expression will be tightly coupled to organism viability. Since the latter is intrinsically linked with the performance of the translation machinery, it is important to understand how rate control exercised by eIF4G over the translation machinery relates to gene expression noise. Since eIF4G1 is the predominant isomer, and the influence of eIF4G2 is equivalent in terms of rate control (see above), the following experiments focus on modulation of eIF4G1 activity alone. We accordingly reengineered the rates of both transcription and translation that direct eIF4G1 synthesis in order to explore the relationship between noise, the intracellular abundance of eIF4G1, and the influence (rate control) of this factor over protein synthesis activity. Guided by previous studies of the relationship between stem-loop stability and translation efficiency (41), we paired P*_TEF1_* with a range of stem-loop structures. For example, this enabled us to identify a combination of the strong P*_TEF1_*promoter with a 5′UTR containing an inhibitory stem-loop structure (M2; Supplementary Figure S5) that generated the same average yield of intracellular eIF4G or of eIF4G::GFP as that produced by the natural *TIF4631* promoter/5′UTR combination (Figure 5A; Supplementary Figure S6). In growth competition experiments, the P*_TEF1_*/M2 *TIF4631* combination was also found to support a growth rate indistinguishable from the equivalent ‘wild-type’ strain (Supplementary Figure S7). In a comparative experiment, we showed that marrying the P*_TEF1_* promoter with *CDC33::GFP* without altering this gene's 5′UTR increased the expression rate ∼5-fold but had only a very small effect on the noise level (Figure 5B; Table 1).

**Figure 6.**
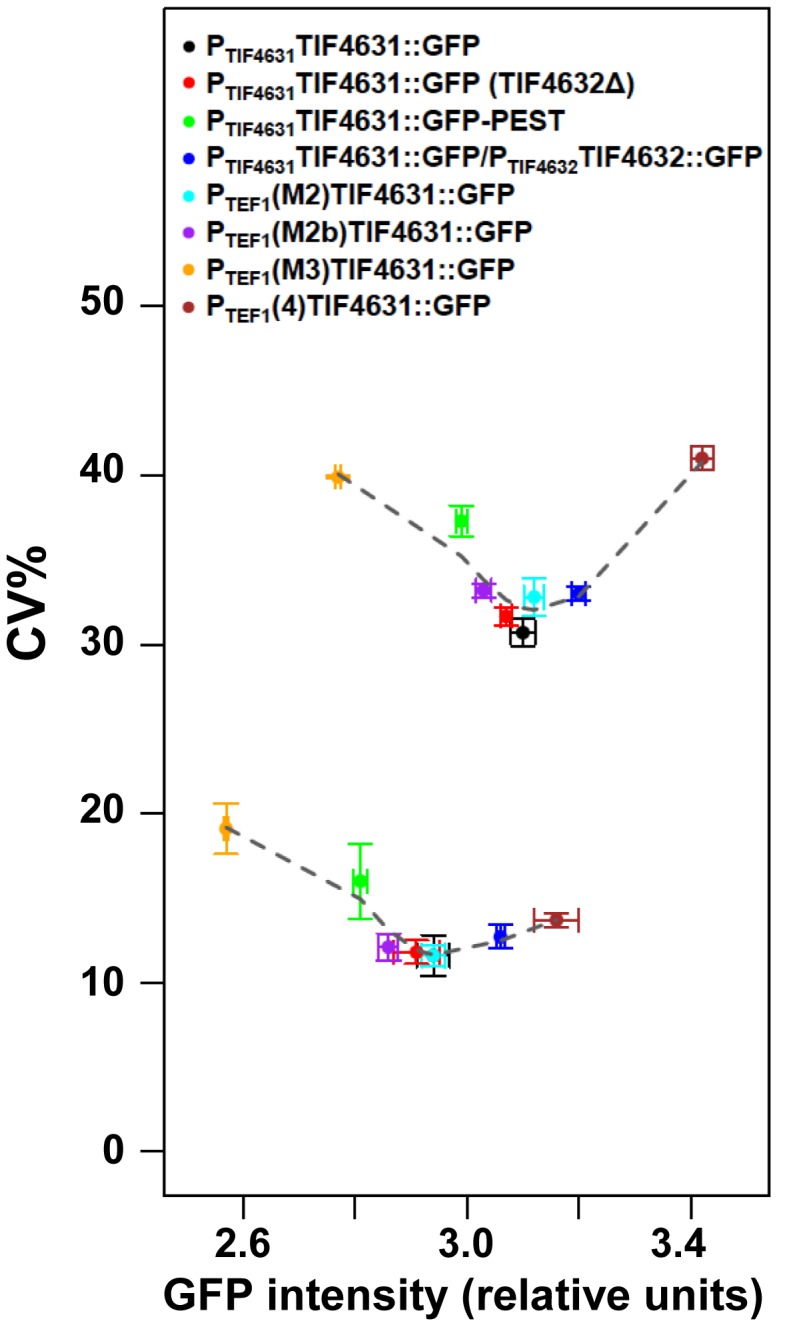
Impact on gene expression noise of modifying the gene expression profile of *TIF4631*. A series of genomic constructs was built that vary the rate of eIF4G1 expression by changing the promoter and/or the 5′UTR. Here we plot the noise for eIF4G1::GFP in the corresponding strains. Data points in the upper plot represent the total population noise of the flow cytometry data for the respective genomic constructs, while those in the lower plot represent the noise values determined via the correction procedure illustrated in Figure 4A, B.

Moreover, by constructing a wider range of genomically integrated constructs that support the synthesis of eIF4G to levels above and below the average wild-type abundance, we were able to paint a broader picture of the relationship between intracellular abundance and noise at the protein level. The resulting curve revealed that the minimum noise level for eIF4G corresponds to the abundance of this factor in wild-type yeast cells (Figure 6), irrespective of whether eIF4G synthesis is driven by P*_TIF4631_* or P*_TEF1_*(M2). In other words, the gene expression noise for eIF4G has evolved to a minimum at the abundance point that also supports maximal protein synthesis and growth (compare Figure 1B). Finally, implementation of a noise modeling tool illustrates that the observed trends cannot be explained by transcriptional noise generation, since such a model predicts that P*_TEF1_*(M2)*TIF4631::GFP*, by virtue of its increased transcript level, should generate less noise than P*_TIF4631_TIF4631::GFP* (Supplementary Figure S8 and ref. 32). Interestingly, the experimentally measured transcriptional noise was indeed reduced for P*_TEF1_*(M2)*TIF4631::GFP* (Figure 2: CV = 0.35, compared to 0.40 for P*_TIF4631_TIF4631::GFP*).

## DISCUSSION

The translation machinery is a primary engine of viability and growth in living cells. Moreover, intrinsic noise in the production of its components can potentially drive extrinsic noise that affects expression of the entire genome. In the present work, we have found that the two isomers of eIF4G manifest barely distinguishable rate control coefficients with respect to global protein synthesis *in vivo*. However, eIF4G1 is the dominant isomer in terms of intracellular protein abundance and corresponding mRNA levels, and the noise characteristics of the two isomers are distinct and weakly correlated. The low abundance, core scaffolding function and distinctive control profile make eIF4G an important subject of study in the context of rate control and gene expression noise. An assembly pathway involving multiple interactions focused around eIF4G is predicted by our computational modeling to have a zone of maximum activity flanked by regions of reduced activity at both lower and higher concentrations. This behavior is neither predicted, nor experimentally observed, for the other cap-binding complex factors. A potential explanation for the drop in protein synthesis associated with eIF4G overproduction derives from the model prediction that an excess of this translation factor distorts the distribution of eIF4G across the respective intermediate complexes in the assembly pathway (see the Supplementary Data; Supplementary Figure S2).

There are many ‘multi-binding-partner’ proteins in complex assembly pathways in living systems and it therefore seems likely that optimal windows of activity analogous to that seen here for eIF4G also feature in a wide range of biochemical systems. It is worth noting that, for the sake of simplicity, we have used a minimal computational model here for the purpose of illustrating the principle that the ‘scaffolding’ role of eIF4G generates a distinctive (although we think unlikely to be unique) type of relationship between abundance and translation machinery activity. It is possible that further refinement of the model (e.g. inclusion of the stimulatory role of eIF4B in assembly of the 48S pre-initiation complex; (26,42,43)) might allow us to achieve even more precise fits to the experimental data.

Analysis of the strains created in this study has demonstrated that the noise level varies across the genomic *TIF4631* constructs that we have engineered, manifesting a minimum that maps to the naturally determined average eIF4G abundance, which also coincides with the point of maximal rate control. Thus minimization of expression noise for this factor has co-evolved with optimization of the rate control it exercises over the translation machinery. The observed relationship of *TIF4631* gene expression noise to changes in intracellular abundance contrasts with the absence of a comparable relationship for *CDC33*. This difference in behavior is likely to be related to the fact that overproduction of eIF4E does not inhibit protein synthesis. Indeed, the observed (intrinsic) protein noise does not correlate with the transcription rate for *TIF4631/2*, but rather with the global protein synthesis rate. Consistent with this correlation, given the marked insensitivity of global protein synthesis to overexpression of *CDC33*, we note that five-times overexpression of this gene does not amplify noise. At the same time, we note our inability to reduce overall gene expression noise for the translation factors eIF4G and eIF4E by increasing transcription rates (and reducing transcript noise) for the individual genes. This could be because these two factors are interdependent components within a subcellular machinery that has evolved to a point of shared minimum noise (a noise ‘floor’, compare ref 40) that is dictated primarily by extrinsic factors. We find that a transcription-based noise generation model does not predict the observed behavior, and therefore detailed further investigation of potential mechanisms will be required in future work. One aspect that deserves particular attention is the observation that replacement of the natural promoter P*_TIF4631_* by the P*_TEF1_* promoter (allied to the stem-loop M2) does not reduce protein-level noise. One possible explanation of this that merits future examination is that such a stem-loop structure might itself in some way act to increase noise.

For genes encoding the components of molecular machinery that is itself part of the expression pathway, the distinction between intrinsic and extrinsic noise becomes blurred. Thus intrinsic noise in the production of a factor such as eIF4G also creates extrinsic noise in the translation of the entire yeast transcriptome, which in turn affects biosynthesis of other machineries, including those for transcription and ribosome biogenesis. Moreover, in any multi-component assembly pathway, the ratio between the participating factors can be as important as the absolute abundance levels of the individual factors. Our measurements indicate that the abundance ratios between the respective cap-binding complex factors are maintained at relatively low values, presumably as the result of selection to minimize inter-component heterogeneity. The coupling between component activity and global noise is seen in the increased noise associated with the use of plasmid-borne genes, and in the fact that noise is also increased when genomic engineering of *TIF4631* expression shifts the eIF4G level either side of the optimal window.

We are not aware of a previous report in the literature of such a convergence of minimal gene expression noise to an optimal rate control point for a subcellular machinery. Nevertheless, our results illustrate how mechanistic constraints are able to dictate behavior of this kind, and it is therefore likely that comparable relationships have also evolved in other biomolecular machineries in living cells. We note that the evolutionary trajectory of a biochemical mechanism can evidently ‘trap’ a core component in a narrow zone of optimal activity but that, despite this constraint, the genetic expression pathway can evolve to minimize noise within the same window. There are, at least in principle, a few different ways that the cell can optimize the relationship between noise and rate control in complex cellular machineries. In one strategy, combining strong transcription and efficient translation to produce a high abundance, even an excess, of a component that has a high rate control coefficient in the near-physiological concentration range (for example, eIF4E or eIF4A) would allow the cell to minimize the impact of abundance fluctuations because these would largely span the relatively abundance-insensitive plateau region. This strategy cannot work for a protein like eIF4G because of its specific abundance response curve and thus it can be hypothesized that noise minimization has had to be optimized to map to a relatively narrow optimal rate control window. An alternative scenario would involve a specific mechanism of intrinsic noise suppression such as autoregulatory feedback or noise filtering via a connected circuit (44). However, we have seen no evidence for the existence of such mechanisms in the case of the cap-complex factors. Finally, these considerations suggest that, in a multi-component biomolecular machine, co-evolution of multiple noise contributions occurs in order to minimize total system noise. In turn, this would suggest that the only potential way to engineer suppression of system noise further might be to reduce the noise for most, if not all, (rate-controlling) components simultaneously. Exploration of this hypothesis would help advance further our understanding of the relationship between noise and evolution.

## References

[1] Duarte N.C., Herrgard M.J., Palsson B.O. (2004). Reconstruction and validation of *Saccharomyces cerevisiae* iND750, a fully compartmentalized genome-scale metabolic model. Genome Res..

[2] Scott M., Klumpp S., Mateescu E.M., Hwa T. (2014). Emergence of robust growth laws from optimal regulation of ribosome synthesis. Mol. Syst. Biol..

[3] Wang Z., Zhang J. (2011). Impact of gene expression noise on organismal fitness and the efficacy of natural selection. Proc. Natl. Acad. Sci. U.S.A..

[4] Gingras A.C., Raught B., Sonenberg N. (1999). eIF4 initiation factors: effectors of mRNA recruitment to ribosomes and regulators of translation. Annu. Rev. Biochem..

[5] Wells S.E., Hillner P.E., Vale R.D., Sachs A.B. (1998). Circularization of mRNA by eukaryotic translation initiation factors. Mol. Cell.

[6] Ptushkina M., von der Haar T., Vasilescu S., Frank R., Birkenhäger R., McCarthy J.E.G. (1998). Cooperative modulation by eIF4G of eIF4E binding to the mRNA 5′cap in yeast involves a site partially shared by p20. EMBO J..

[7] Gross J.D., Moerke N.J., von der Haar T., Lugovskoy A.A., Sachs A.B., McCarthy J.E.G., Wagner G. (2003). Ribosome loading onto the mRNA cap is driven by conformational coupling between eIF4G and eIF4E. Cell.

[8] Aitken C.E., Lorsch J.R. (2012). A mechanistic overview of translation initiation in eukaryotes. Nat. Struct. Mol. Biol..

[9] de la Cruz J.I., Iost I., Kressler D., Linder P. (1997). The p20 and Ded1 proteins have antagonistic roles in eIF4E-dependent translation in *Saccharomyces cerevisiae*. Proc. Natl. Acad. Sci. U.S.A..

[10] Senissar M., Le Saux A., Belgareth-Touzé N., Banroques J., Tanner N.K. (2014). The DEAD-box helicase Ded1 from yeast is an mRNP cap-associated protein that shuttles between the cytoplasm and nucleus. Nucleic Acids Res..

[11] Firczuk H., Kannambath S., Pahle J., Claydon A., Beynon R., Duncan J., Westerhoff H., Mendes P., McCarthy J.E.G. (2013). An *in vivo* control map for the eukaryotic mRNA translation machinery. Mol. Sys. Biol..

[12] Howard A., Rogers A.N. (2014). Role of translation initiation factor 4G in lifespan regulation and age-related health. Ageing Res. Rev..

[13] Bardwell L. (2005). A walk-through of the yeast mating pheromone response pathway. Peptides.

[14] Fatica A., Tollervey D. (2002). Making ribosomes. Curr. Opin. Cell Biol..

[15] Kaneko T., Murata S. (2012). Using siRNA techniques to dissect proteasome assembly pathways in mammalian cells. Methods Mol. Biol..

[16] MacLean B., Tomazela D.M., Shulman N., Chambers M., Finney G.L., Frewen B., Kern R., Tabb D.L., Liebler D.C., MacCoss M.J. (2010). Skyline: an open source document editor for creating and analyzing targeted proteomics experiments. Bioinformatics.

[17] Rahman S., Zenklusen D. (2013). Single-molecule resolution fluorescent *in situ* hybridization (smFISH) in the yeast *S. cerevisiae*. Methods Mol. Biol..

[18] Raser J.M., O'Shea E.K. (2004). Control of stochasticity in eukaryotic gene expression. Science.

[19] Harris L.A., Hogg J.S., Tapia J-J., Sekar J.A.P., Gupta S., Korsunsky I., Arora A., Barua D., Sheehan R.P., Faeder J.R. (2016). BioNetGen 2.2: advances in rule-based modelling. Bioinformatics.

[20] Hoops S., Sahle S., Gauges R., Lee C., Pahle J., Singhal M., Xu L., Mendes P., Kummer U. (2006). COPASI - a COmplex PAthway SImulator. Bioinformatics.

[21] Hooke R., Jeeves T.A. (1961). “Direct search" solution of numerical and statistical problems. J. Assocn. Comput. Mach..

[22] Kennedy J., Eberhart R. (1995). Particle Swarm Optimization. Proc Fourth IEEE International Conference on Neural Networks.

[23] Clarkson B.K., Gilbert W.V., Doudna J.A. (2010). Functional overlap between eIF4G isoforms in *Saccharomyces* cerevisiae. PLoS ONE.

[24] Lang V., Zanchin N.I., Lünsdorf H., Tuite M., McCarthy J.E.G. (1994). Initiation factor eIF-4E of *Saccharomyces cerevisiae*: Distribution within the cell, binding to mRNA, and consequences of its overproduction. J. Biol. Chem..

[25] Hilliker A., Gao Z., Jankowsky E., Parker R. (2011). The DEAD-box protein Ded1 modulates translation by the formation and resolution of an eIF4F-mRNA complex. Mol. Cell.

[26] Park E.H., Walker S.E., Zhou F., Lee J.M., Rajagopal V., Lorsch J.R., Hinnebusch A.G. (2013). Yeast eukaryotic initiation factor 4B (eIF4B) enhances complex assembly between eIF4A and eIF4G in vivo. J. Biol. Chem..

[27] von der Haar T, Oku Y., Ptushkina M., Moerke N., Wagner G., Gross J.D., McCarthy J.E.G. (2006). Folding transitions during assembly of the eukaryotic mRNA cap-binding complex. J. Mol. Biol.

[28] Kapp L.D., Lorsch J.R. (2004). The molecular mechanics of eukaryotic translation. Annu. Rev. Biochem..

[29] Hinnebusch A.G., Lorsch J.R. (2012). The mechanism of eukaryotic translation initiation: new insights and challenges. Cold Spring Harb. Perspect. Biol..

[30] Heidelberger M., Kendall F.E. (1929). A quantitative study of the precipitin reaction between type III Pneumococcus polysaccharide and purified homologous antibody. J. Exp. Med..

[31] Bray D., Lay S. (1997). Computer-based analysis of the binding steps in protein complex formation. Proc. Natl. Acad. Sci. U.S.A..

[32] Kœrn M., Elston T.C., Blake W.J., Collins J.J. (2005). Stochasticity in gene expression: from theories to phenotypes. Nat. Rev. Gen..

[33] Holstege F.C., Jennings E.G., Wyrick J.J., Lee T.I., Hengartner C.J., Green M.R., Golub T.R., Lander E.S., Young R.A. (1998). Dissecting the regulatory circuitry of a eukaryotic genome. Cell.

[34] Zenklusen D., Larson D.R., Singer R.H. (2008). Single-RNA counting reveals alternative modes of gene expression in yeast. Nat. Struct. Mol. Biol..

[35] Taniguchi Y., Choi P.J., Gene-Wei L., Chen H., Babu M., Hearn J., Emili A., Xie X.S. (2010). Quantifying *E. coli* proteome and transcriptome with single-molecule sensitivity in single cells. Science.

[36] Salari R. (2012). Teasing apart translational and transcriptional components of stochastic variations in eukaryotic gene expression. PLoS Comput. Biol..

[37] Elowitz M.B., Levine A.J., Siggia E.D., Swain P.S. (2002). Stochastic gene expression in a single cell. Science.

[38] Zopf C.J., Quinn K., Zeidman J., Maheshri N. (2013). Cell-cycle dependence of transcription dominates noise in gene expression. PLoS Comput. Biol..

[39] Kempe H., Schwabe A., Crémazy F., Vershure P.J., Bruggeman F.J. (2015). The volumes and transcript counts of single cells reveal concentration homeostasis and capture biological noise. Mol. Biol. Cell.

[40] Newman J.R., Ghaemmaghami S., Ihmels J., Breslow D.K., Noble M., DeRisi J.L., Weissman S. (2006). Single-cell proteomic analysis of *S. cerevisiae* reveals the architecture of biological noise. Nature.

[41] McCarthy J.E.G. (1998). Posttranscriptional control of gene expression in yeast. Microbiol. Mol. Biol. Rev..

[42] Harms U., Andreou A.Z., Gubaev A., Klostermaier D. (2014). eIF4B, eIF4G and RNA regulate eIF4A activity in translation initiation by modulating the eIF4A conformational cycle. Nucleic Acids Res..

[43] Sen N.D., Zhou F., Harris M.S., Ingolia N.T., Hinnebusch A.G. (2016). eIF4B stimulates translation of long mRNAs with structured 5’UTRs and low closed-loop potential but weak dependence on eIF4G. Proc. Natl. Acad. Sci. U.S.A..

[44] Chalancon G., Ravarani C.N.J., Balaji S., Martinez-Aria A., Aravind L., Jothi R., Babu M.M. (2012). Interplay between gene expression noise and regulatory network architecture. Trends Genet..

